# Primary Cutaneous Anaplastic Large Cell Lymphoma of the Oral Cavity Successfully Treated with Brentuximab Vedotin

**DOI:** 10.1155/2019/9651207

**Published:** 2019-09-17

**Authors:** Federico Meconi, Roberto Secchi, Raffaele Palmieri, Sara Vaccarini, Vito Mario Rapisarda, Laura Giannì, Fabiana Esposito, Ida Provenzano, Daniela Nasso, Livio Pupo, Maria Cantonetti

**Affiliations:** Department of Biomedicine and Prevention, Haematology Division, University of Tor Vergata, Viale Oxford 81, Rome, Italy

## Abstract

Primary cutaneous anaplastic large cell lymphoma is a CD-30 positive lymphoproliferative disorder with good prognosis, usually treated with radiation therapy and surgery. Head, neck, and extremities are the most frequently involved sites. In this paper, we describe an unusual case of oral localization, recurring after skin-involving radiotherapy, successfully treated with sixteen cycles of brentuximab vedotin. This could be a more effective approach with a less detrimental toll for treating these rare disorders.

## 1. Introduction

Primary cutaneous CD-30 positive lymphoproliferative disorders are a rare and heterogeneous group of primary skin tumors, accounting for about 30% of all cases of primary cutaneous T-cell lymphomas (PCTCL) [1]. The group comprises primary cutaneous anaplastic large cell lymphoma (PCALCL), lymphomatoid papulosis (LyP), and borderline cases.

PCALCL is most commonly found in adults (median age at diagnosis is 60 years old), occurring more often in men than women (3 : 1). Macroscopically, PCALCL usually presents itself with red to violaceous lesions, rapidly growing and frequently ulcerative. Head, neck, and extremities are the most frequently involved sites, both for solitary lesions or for multiple grouped nodules. Systemic symptoms, such as fever, fatigue, and mucosal involvement, are rare in PCALCL. However, in at least 10% of cases, neoplastic CD30+ cells can be found in locoregional lymph nodes. Its course is generally indolent (dissemination is uncommon); spontaneous regression occurs in about 40% of cases, even though partial remission is documented more frequently than complete ones [2–4].

The first line treatment currently accepted is radiation therapy or surgical removal, whenever possible. In case of multifocal lesions, methotrexate is accepted followed by low-dose maintenance [1]. However, brentuximab vedotin (BV) was recently included in the National Comprehensive Cancer Network Guidelines as a first line treatment. Recurrences are common in both the same or in different cutaneous sites. PCALCL carries a favorable prognosis with a 5-year survival rate over 90% [5].

Histopathologically and microscopically, PCALCL exhibits a high expression rate of CD30+ antigen (CD30 positivity is required for diagnosis in at least 75% of atypical cells), positivity for CD4+ antigen and does not present ALK positivity [6]. In this article, we describe a rare case of PCALCL of the hard palate that was successfully treated with BV.

## 2. Case Report

In November 2016, a 40-year-old Caucasian male came to our attention for a nodular, erythematous, nonulcerated, well-defined, and indurated lesion localized in his right forearm.

A skin punch biopsy of the lesion was performed, showing an inflammatory multinodular infiltration of lymphohistiocytic cells in both deep and superficial dermis, including many large, atypical, anaplastic cells with frequent mitosis, characterized by the following immunohistochemistry: CD45+, CD2+, CD3+/–, CD7+/–, CD20–, CD30+, and ALK–. To rule out systemic anaplastic large cell lymphoma, we performed a bone marrow biopsy (negative for malignant cells) and a CT/PET scan that revealed a significant and singular uptake of ^18^F-FDG in the right forearm region (matching precisely the tumor localization). As we made diagnosis of PCALCL, the patient began radiation therapy (dose of 3600 cGy), achieving complete response.

A new nodular erythematous lesion of 1 cm appeared in March 2017 in the right inguinal region and was unsuccessfully treated with topical steroids. One month later, when it expanded up to 3 cm, a new CT/PET scan was performed and revealed the lesion as the only pathological uptake in the right inguinal region. The patient underwent another radiation therapy of 3600 cGy, obtaining complete response again.

**Figure 1:**
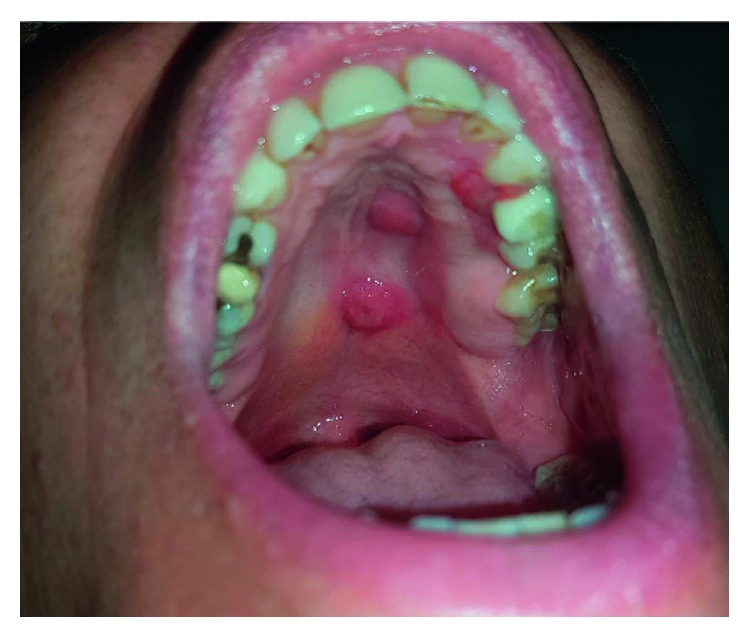
Oral cavity before therapy with BV.

In May 2017, two more nodular lesions appeared in the patient's oral cavity, in the left hard palate (Figure 1). A punch biopsy showed histopathological and immunohistochemical findings consistent with PCALCL. As the lesions did not show spontaneous regression in one month of clinical observation and the patient was experiencing oral discomfort in eating and chewing, it was decided to proceed with treatment.

**Figure 2:**
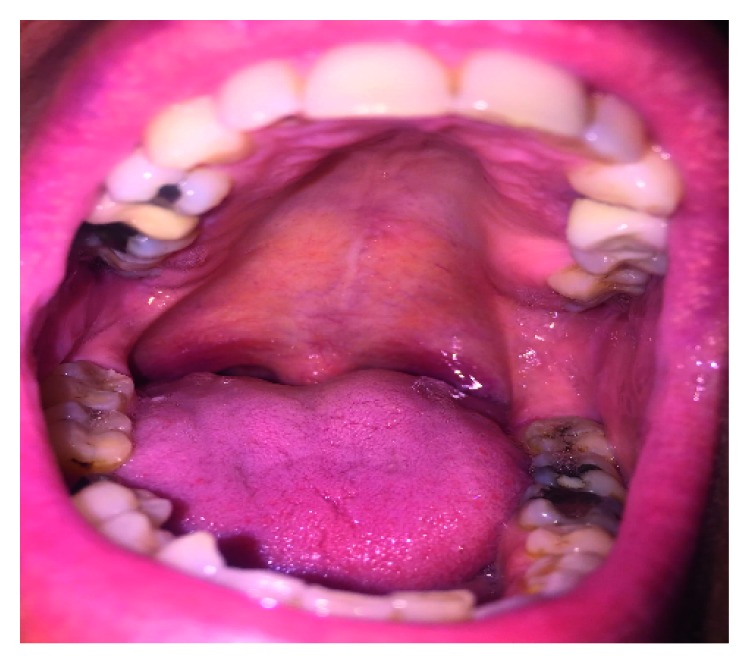
Oral cavity after the 2nd cycle of BV.

To avoid radiation therapy and its side effects and taking into account the short time to relapse that the patient experienced with previous radiation therapy in different sites, it was decided to start systemic immunochemotherapy with brentuximab vedotin (BV) 1.8 mg/Kg every 21 days. Interesting later reviews about reduction in doses and numbers of cycles of BV have been published recently [7], showing these protocols could be as effective as the standard therapy. After only two cycles of BV, the patient showed complete regression of nodular lesions with reappearance of normal mucosa of the oral cavity (Figure 2). The final CT/PET scan evaluation was negative for any disease localization.

Since the patient did not present any neuropathy and experienced early relapse after initial remission obtained with radiotherapy, we continued treatment with BV for sixteen cycles [8] with no reduction in dose. All sixteen cycles were performed since we could not find any data showing that the reduction of cycles could lead to the same disease-free survival, trying to ensure our patient the longest possible complete remission.

## 3. Conclusions

To the best of our knowledge, this is the first documented case of PCALCL present only in the hard palate, while other few cases of similar processes involving mucosal sites of the head and neck have been previously reported with one case of systemic anaplastic large cell lymphoma involving the hard palate [9].

We demonstrated that oral PCALCL could be successfully treated with systemic immune-chemotherapy with brentuximab vedotin, an anti-CD30 antibody conjugated to monomethyl auristatin E and an effective antimicrotubule agent that was previously approved for Hodgkin lymphoma, ALK-positive anaplastic large cell lymphoma [10], and other cutaneous lymphoproliferative disorders, such as mycosis fungoides.

Using immune-chemotherapy instead of radiation therapy can prevent several side effects, such as mucositis, candidosis, dysgeusia, radiation caries, osteoradionecrosis, soft tissue necrosis, progressive periodontal attachment loss, trismus, and xerostomia, which are all well documented while treating mucosal lesions with radiations [11].

Brentuximab's most common side effect is peripheral sensitive neuropathy that can lead to dose reduction or even drug withdrawal. However, our patient did not experience such neuropathy and could complete therapy within the sixteen cycles at full dose.

Due to the rarity of this group of cutaneous lymphoproliferative disorders (even rarer if we consider those found in the oral cavity), it is still a challenge to make a trial comparing radiation therapy with immune-chemotherapy using BV. From this case, we can infer that although BV is not side-effect free, it offers a safe choice to treat primary cutaneous CD-30 positive lymphoproliferative disorders no longer responding to radiation therapy and standard chemotherapy or cases where the anatomic site involved does not allow RT or must be avoided due to its side effects.
